# Behavior change techniques to reduce sedentary behavior and increase physical activity in people at risk for cardiovascular disease: a scoping review

**DOI:** 10.3389/fspor.2026.1639584

**Published:** 2026-02-09

**Authors:** Chen Wu, Qi Zhou, YuTing Yang, Lili Yang

**Affiliations:** 1 Department of Nursing, The Fourth Affiliated Hospital of School of Medicine, and International School of Medicine, International Institutes of Medicine, Zhejiang University, Yiwu, China; 2 Nursing Department, Sir Run Run Shaw Hospital, Zhejiang University School of Medicine, Hangzhou, China; 3 Hangzhou Linping District Hospital of Integrated Traditional Chinese and Western Medicine, Hangzhou, China

**Keywords:** behavior change techniques, cardiovascular disease, intervention, physical activity, risk, sedentary behavior

## Abstract

**Introduction:**

Most people at high risk of cardiovascular disease have high levels of sedentary behavior and little physical activity, which increases the incidence of cardiovascular disease. Although there have been many studies confirming the benefits of reducing sedentary behavior and increasing physical activity, the effectiveness and sustainability of the interventions have been limited. Behavior Change Techniques are the smallest unit of effect in the process of changing behavior. Behavior Change Techniques Taxonomy v1 is the first consensus-based interdisciplinary taxonomy of Behavior Change Techniques to date. This can help researchers identify effective components of behavior change interventions and transform general interventions into targeted interventions, and improve intervention effectiveness.

**Aims:**

This scoping review aimed to identify and evaluate the behavior change interventions to reduce sedentary behavior and increase physical activity in people at high risk for cardiovascular disease and to improve the effectiveness of interventions.

**Methods:**

We conducted a scoping review in accordance with the Arksey and O'Malley framework. Eleven databases were searched (BMJ Best Clinical Practice, UpToDate, Cochrane Library, EMBase, PubMed, Web of Science, China National Knowledge Infrastructure, Wanfang Data, Chinese Biomedical Database, VIP, Yimaitong Database) from inception through 20 July, 2025, following the scoping review methodology. We synthesized the interventions narratively using the Behavior Change Techniques Taxonomy v1.

**Results:**

Nineteen articles were ultimately included, including five guidelines, seven randomized controlled trials (RCTs), three cross-sectional studies, one quasi-experimental study, one qualitative study, one cohort study and one systematic review. Categorized according to the Behavior Change Techniques Taxonomy v1, it contains a total of eleven aspects, including goals and planning, feedback and monitoring, social support, shaping knowledge, natural consequences, comparison of behaviour, repetition and substitution, regulation, antecedents, identity, self-belief.

**Conclusions:**

Previous studies did not cover the persistence of intervention effects, and there is still controversy regarding the cardiovascular effects of different physical activity types. In future, a more comprehensive approach should be adopted to intervene in behaviors, such as physical activity types, cultural backgrounds, and combined with behavior change theories and Behavior Change Techniques, to implement targeted interventions for behaviors.

## Strengths and limitations of the review

- Summarizes interventions for SB and PA in people at high risk of cardiovascular disease.
- Providing a basis for future management of people at high risk of cardiovascular disease.
- This protocol was not registered previously.
- Non-English and Chinese language articles were excluded.

## Introduction

Cardiovascular disease is a collective term for diseases of the heart and blood vessels. It generally refers to ischaemic or haemorrhagic diseases that occur in the heart, brain and tissues throughout the body, including coronary heart disease, myocardial infarction, stroke and peripheral vascular disease (1). The World Health Organization (WHO) states that cardiovascular disease is the most common cause of death worldwide, accounting for half of all deaths from non-communicable diseases (2). Studies have shown that as many as 10% people in China are at high risk of cardiovascular disease, and the number is still rising (3). Thus, it is necessary to effectively manage people at high risk of cardiovascular disease and reduce the incidence of cardiovascular disease.

Sedentary behavior (SB) refers to behaviour in the waking state while sitting or lying down with an energy expenditure of less than or equal to 1.5 metabolic equivalents, including activities such as watching TV, reading, and using a computer (4). Studies have found that prolonged SB can cause blood flow to slow down, generate a large amount of reactive oxygen species that damage vascular endothelial function, and increase the risk of cardiovascular disease (5). For every additional hour of sedentary behaviour per day, the risk of hypertension increases by 4% (6). The risk of death is 1.48 and 2.92 respectively for 10 h and 12 h of SB per day (7). Meanwhile, Ekelund et al. (8) found that reducing sedentary behaviour and increasing moderate-to-vigorous physical activity can reduce mortality from cardiovascular disease. The WHO previously only emphasized the importance of the duration and frequency of physical activity, but in the 2020 guidelines on physical activity and sedentary behaviour, new recommendations have been specifically updated for reducing SB for all age groups, pregnant women, people with chronic diseases and people with disabilities (3). SB has been identified as an independent risk factor for cardiovascular disease, which provides a new entry point for health management for people at high risk of cardiovascular disease. Therefore, reducing sedentary behavior and increasing physical activity in people at risk for cardiovascular disease can effectively reduce the incidence of cardiovascular disease and improve people's quality of life.

Physical activity (PA) is a critical factor to improve cardio metabolic health. Health benefits are associated with following at least 150 min of moderate physical activity or 75 min of vigorous physical activity per week. Furthermore, the prevention of weight gain is prominent when physical activity is performed at ≥three metabolic equivalents for >150 min per week (9). Some researchers have suggested that risk can be further reduced by increasing physical activity, especially moderate-intensity physical activity, in individuals at high risk for cardiovascular disease (10). These findings underscore the need for personalized physical activity recommendations tailored to those at risk for cardiovascular disease.

A Behavior Change Techniques (BCTs) is an observable, replicable measure, which directly applies to both the target population and behavior (11). Identification of BCTs in heterogeneous interventions allows analyzing which common BCTs are associated with effective outcomes (12). The Behavior Change Techniques Taxonomy v1(BCTTv1) is a reliable and valid method for synthesizing the content of interventions as it labels and comprehensively describes 93 BCTs potentially applied in interventions (12). However, the current research on BCTs remain fragmented, with many studies only focusing on selected aspects of the framework (13). This scoping review aimed to synthesize and evaluate the behavior change interventions to reduce sedentary behavior and increase physical activity in people at risk for cardiovascular disease through the BCTTv1 by Michie et al. (12). This can help researchers identify effective components of behavior change interventions and transform general interventions into targeted interventions, and improve intervention effectiveness.

### Objectives of the review

The benefits of SB and PA interventions targeting populations at high risk for cardiovascular disease have been demonstrated, but the effectiveness and sustainability of the interventions have been limited. This scoping review aimed to identify and evaluate the behavior change techniques to reduce sedentary behavior and increase physical activity in people at high risk for cardiovascular disease. This result may trigger future research to develop more targeted and effective interventions to reduce sedentary behavior and increase physical activity in people at risk for cardiovascular disease.

## Materials and methods

This study utilized a scoping review methodology to comprehensively synthesize the most effective approaches for intervening in the SB and promoting PA among individuals at high risk of cardiovascular disease. The review was conducted following the five-stage methodological framework proposed by Arksey and O'Malley, which consists of: (1) identifying the research question; (2) identifying relevant studies; (3) selecting studies; (4) charting the data; and (5) collating, summarizing, and reporting the results. The reporting of this review followed the Preferred Reporting Items for Systematic Reviews and Meta-Analyses Extension for Scoping Reviews (PRISMA-ScR) recommendations (14).

### Stage 1: identifying the research question

The research question that guided this review was: “What are the interventions to reduce SB and promote PA among individuals at risk of cardiovascular disease?”

### Stage 2: identifying relevant studies

We searched eleven databases, including BMJ Best Clinical Practice, UpToDate, Cochrane Library, EMBase, PubMed, Web of Science, China National Knowledge Infrastructure, Wanfang Data, Chinese Biomedical Database, VIP, Yimaitong Database, from inception to 20 July, 2025. The key concepts were used: “Cardiovascular Disease”, “High Risk OR Risk Factors”, and “Sedentary Behavior OR Movement OR Physical Exercise OR Physical Activity OR Exercise”.

### Stage 3: selecting studies

Literature inclusion criteria for this review were: (1) The study subjects were aged ≥20 years and defined as a high-risk population for cardiovascular disease; (2) studies on SB or PA interventions; (3) the evidence type was clinical decision-making, guidelines, best clinical practice, systematic reviews, evidence summaries, expert consensus, and Randomized Controlled Trials (RCTs); (4) the publication language was Chinese and English. Exclusion criteria were: (1) full text not available; (2) low quality assessment of literature; (3) duplicate publication; (4) interpretation/translation/updated version of guidelines or consensus; (5) incomplete content such as drafts/abstracts/conferences.

For summaries of the included evidence, the original literature of the recommendations was evaluated. Guidelines were evaluated using the Appraisal of Guidelines for Research and Evaluation II (AGREE II), a 2017 update of the UK's clinical guidelines research and evaluation system. The JBI Center for Evidence-Based Health Care RCT Evaluation Tool (2016), Qualitative Research Evaluation Tool (2016), Cross-sectional Research Evaluation Tool (2016), Experimental-like Research Evaluation Tool (2016), Cohort Study Evaluation Tool (2016) and Systematic Evaluation Tool (2016) were used to evaluate the methodological quality of the RCTs, qualitative studies, cross-sectional studies, experimental-like research, cohort study and systematic evaluation.

### Stage 4: charting the data

The abstracted data included author(s), year of publication, country, type of study, main findings. The first draft of the data charts of five randomly selected studies was completed independently by two reviewers (YTY and CW). The chart form was revised through discussion among the research team to extract information from all the included studies. The data were extracted by two authors and checked by another author (QZ). Any disagreements were resolved by discussion among the whole team.

### Stage 5: collating, summarizing, and reporting the results

For the coding of behavioral intervention content, two reviewers who had completed prior online training independently performed the coding for the included studies. All 93 BCTs outlined in the BCTTv1 were considered for each study, with both reviewers explaining their rationale for assigning the BCT in each instance. If a consensus could not be reached, the proposed BCT was not included.

### Patient and public involvement

Patients and/or the public were not involved in the design, or conduct, or reporting or dissemination plans of this research.

## Results

### Study selection

**Figure 1:**
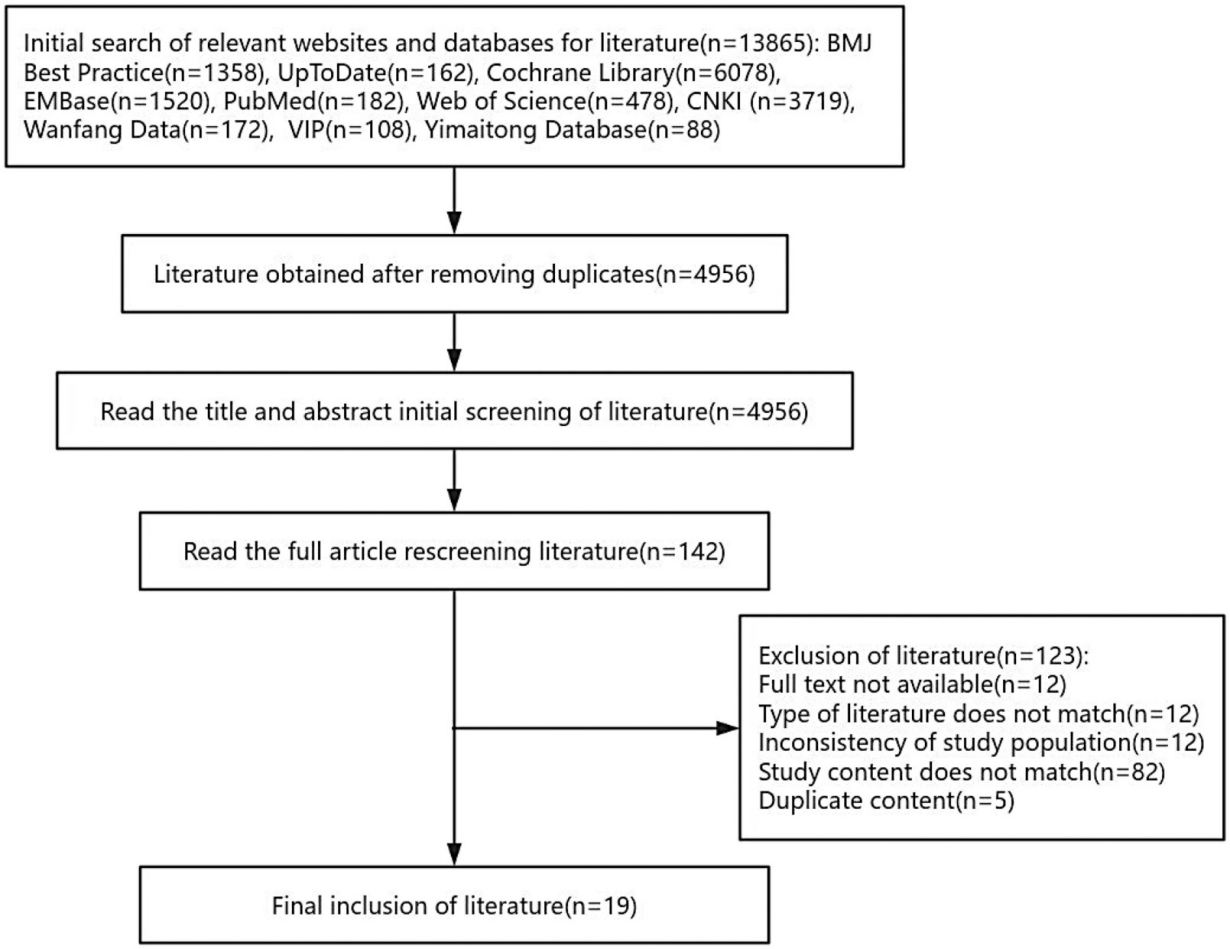
PRISMA flowchart showing a selection of articles for scoping review.

A total of 13,865 studies were searched through the database, and 4,956 remained after removing duplicate studies. According to the inclusion and exclusion criteria, 142 studies were reminded after screening the titles and abstracts for full-text reading, of which 123 studies were excluded due to the following reasons: full text not available, type of literature does not match, inconsistency of study population, study content does not match, duplicate content. Therefore, 19 studies were finally included and the literature screening process is shown in Figure 1.

### Methodogical quality

The overall quality of the 19 included studies was moderate, which were described as follows.

The average scores of the five guideline quality evaluations in each field were all above 70% (15–19), and thus they were approved for inclusion. A total of 7 RCTs were included in this study (20–26). Two studies (20, 21) evaluated the item 2 “whether the study subjects were blinded or not” as unclear. Four studies (20–22, 26) evaluated the item 3 “whether allocation concealment was used for grouping” as unclear. Five studies evaluated (21, 22, 24–26) the item 5 “whether outcome evaluators were blinded” as unclear. One study (23) was negative for items 2 and 3. The evaluation results of other items were all yes, and were approved for inclusion.

This study included a total of 3 cross-sectional studies (27–29). In one of the studies (27), the evaluation result for item 2 “How was the research population selected?” was unclear. In the other two studies (27, 28), the evaluation results for item 3 “Did the inclusion and exclusion criteria for the sample clearly describe them?” were negative. The evaluation results of other items were all yes, and were approved for inclusion.

A total of 1 systematic review was included in this study (30), and the question of item 8, “Is the method of synthesizing/combining studies appropriate?” The evaluation result was unclear. The evaluation results of other items were all yes, and were approved for inclusion.

A total of 1 cohort study was included in this study (31), and all items were evaluated as yes, which was approved for inclusion.

A total of 1 quasi-experimental study (32) and 1 qualitative study (33) were included in this study. All items were evaluated as yes and were approved for inclusion.

### Characteristics of the included studies

19 studies included in this scoping review were published between 2003 and 2023. The countries in which studies were conducted were USA (*n* = 7) (17, 19, 24, 26, 28, 29, 32), the UK (*n* = 5) (21, 22, 31), China (*n* = 3) (15, 16, 18), Netherlands (*n* = 1) (27), Australia (*n* = 1) (20), Iran (*n* = 1) (23), Korea (*n* = 1) (25). The types of studies included guidelines (*n* = 5), RCTs (*n* = 7), cross-sectional studies (*n* = 3), quasi-experimental study (*n* = 1), cohort study (*n* = 1),（qualitative study (*n* = 1), systematic review (*n* = 1).

Across the 19 studies Based on the BCCTv1 (12), a total of 11 distinct BCTs were identified. The most frequently employed BCTs were “Comparison of behaviour”, “ Repetition and substitution”, “ antecedents”, “Five BCT groupings were not found in any study:” “Associations”, “Comparison of outcomes”, “Reward and threat”, “Scheduled consequences”, “Covert learning”.

A comprehensive breakdown of the coded BCTs for each intervention can be found in Table 1.

**Table 1:** Comprehensive breakdown of the coded BCTs for interventions.

Intervention	The number of included studies supporting the intervention	Article citation
1. Goals and planning	7	
1.1 Incorporating physical activity into the vital signs category	1	(28)
1.2 Assessing the health status of individuals	2	(15, 17)
1.3 Assessing the risk profile of individuals	1	(18)
1.4 Physical activity assessment and counseling in medical facilities	3	(15, 17, 18)
2. Feedback and monitoring	2	
2.1 Utilization of mobile applications	1	(25)
2.2 Interventionists monitor the accuracy of behavior change measures throughout the process	1	(32)
3. Social support	7	
3.1 Support from peers and family members	1	(33)
3.2 Ongoing assessment and support for interveners	1	(32)
3.3 Health worker support in medical institutions	4	(15, 17, 27, 28)
3.4 Community staff support	1	(32)
4. Shaping knowledge	10	
4.1 Implementing theory- and evidence-based health promotion activities	1	(32)
4.2 Swing sports: badminton, table tennis, etc.	2	(15, 29)
4.3 Aerobic exercise: cycling, running, aerobics, etc.	2	(15, 29)
4.4 Resistance exercises: dumbbells, push-ups, squats, machine weights and resistance bands	4	(15, 16, 18, 19)
4.5 Others:Bone-building exercises, balance exercises or flexibility exercises, such as tai chi, yoga, etc.	1	(15)
5. Natural consequences	1	
5.1 Cardiovascular diseases: coronary heart disease, hypertension, heart failure, arrhythmia, stroke, peripheral artery disease.	1	(15)
6. Comparison of behaviour	14	
6.1 Exercise for 30 min 3 times a week at a heart rate of 70%–80% of your maximum heart rate.	4	(15–18)
6.2 At least 30 min of moderate-intensity physical activity every day, 5 days a week (at least 150 min/week)	4	(15–18)
6.3 15 min a day, 5 days a week, high-intensity physical activity (at least 75 min/week)	2	(16, 19)
6.4 For healthy adults, 8–10 different resistance exercises can be selected, each performed in 1–3 sets with moderate intensity loads, allowing 8–12 repetitions per set to achieve volitional fatigue, ≥2 times per week. PA for the same muscle group should be separated by at least 1 day.	4	(15–18)
6.5 vigorous intermittent lifestyle physical activity	1	(31)
7. Repetition and substitution	14	
7.1 Interruption of continuous sedentary activity for 30 min for 1 min of high-intensity PA with a heart rate of 90% of maximal heart rate	1	(21)
7.2 Interruption of continuous sedentary behavior for 30 min to perform 2 min of PA	1	(22)
7.3 Interruption of continuous sedentary activity for 1 h for 8 min of moderate-intensity PA	1	(26)
7.4 20 min of high-intensity PA 45 min after a meal, followed by a long period of SB	1	(21)
7.5 The basic goal for adults is to increase PA and decrease SB	4	(15–17, 20)
7.6 Changing SB to standing	2	(18, 20)
7.7 Changing SB to light physical activity	1	(16)
7.8 Changing sedentary behavior to moderate to high intensity physical activity	3	(15, 16, 26)
8. Regulation	4	
8.1 PA can be combined with daily lifestyle	1	(15)
8.2 Combining running and cycling has a synergistic effect	1	(29)
8.3 The combination of running and aerobic PA has a synergistic effect	1	(29)
8.4 The combination of running and racquetball has a synergistic effect	1	(29)
9. Antecedents	8	
9.1 Changing the workplace environment	4	(20, 25, 30, 32)
9.2 Changing community environments	1	(32)
9.3 Prescribing physical activity in medical facilities	3	(15, 17, 18)
10. Identity	2	
10.1 Improvement of self-perception	2	(24, 25)
11. Self-belief	1	
11. 1Intensive behavior change training	1	(32)

### Goals and planning

Seven studies involve goals and planning, this includes an assessment of the health status (15, 17), PA history (18), and possible risk profile of the person at risk for cardiovascular disease (18). It is recommended that physical activity assessment and counseling be implemented in healthcare settings and that physical activity be included as one of the vital signs (28). Detailed assessments can identify discrepancies between current behaviors and the target behaviors set, leading to the development of targeted implementation plans. At the same time, a written code of conduct is developed, witnessed by others, that requires the subject of the intervention to acknowledge and commit to behavioral change.

### Feedback and monitoring

Two studies involve feedback and monitoring. With the development of technology, sedentary time, frequency and intensity of PA can be recorded with the help of mobile applications for the study subjects (25). The interventionist as a good monitor should give hints or evaluations based on the performance of the study subjects (32). Prompts and feedback were given to the study participants based on their implementation, thus ensuring that the intervention was implemented correctly and effectively.

### Social support

Seven studies involve social support. Support for the intervener is also important during the intervention process, with the intervener monitoring the implementation of behavioral changes throughout the process and providing support and assistance when anomalies arise (32). Depending on the intervention site, the person supervising may change. The person supporting in the hospital may be a healthcare worker (33), the person supporting in the community may be a community worker (32), the person supporting at home may be a family member (33), and the person supporting in the workplace may be a coworker (20, 25, 30, 32). Of course in addition to behavioral support, emotional encouragement is equally important. Effective support allows individuals to be more confident and motivated in the change process.

### Shaping knowledge

Ten studies involve shaping knowledge. Knowledge of the intervention process should be gained through the collection and interpretation of data, so interventions should be carried out based on evidence and theory (32). Of course knowledge can also be derived from relevant guidelines or expert consensus, which recommend types of exercise during PA, such as swing type exercises: badminton, table tennis, etc (15, 29), aerobic exercises: cycling, running, aerobics, etc (15, 29), resistance exercises: dumbbells, push-ups, squats, machine weights and resistance bands, etc (15, 16, 18, 19), and others: bone-enhancing type exercises, balance-type exercises, or flexibility exercises such as tai chi and yoga (32). Of course the whole process of knowledge formation should be integrated with the social and environmental context, emotions, and perceptions of the research subject to jointly reach a scientifically valid consensus.

### Natural consequences

One study involves natural consequences. If people at risk for cardiovascular disease don't change their bad habits, continued sedentary behavior and lack of PA are likely to lead to cardiovascular disease, such as coronary heart disease, hypertension, heart failure, arrhythmia, stroke, peripheral artery disease (15). which may eventually lead to death (16). So if the intervention is followed up, we can paste pictures of cerebral infarction into public places or make advertisements to warn the public. We can inspire feelings of regret in people at high risk for cardiovascular disease who subsequently experience adverse outcomes if no change occurs.

### Comparison of behaviour

Fourteen studies involve comparison of behaviour. Current guidelines in various countries recommend the frequency and intensity of weekly exercise. 30 min of PA 3 times a week, with a heart rate of 70%–80% of maximum heart rate (25). At least 30 min of moderate-intensity PA every day, 5 days a week (at least 150 min/week) (15–18). 15 min of high-intensity PA every day, 5 days a week (at least 75 min/week). For healthy adults, choose 8–10 different resistance exercises, perform each exercise with 1–3 sets of moderate intensity loads, allow 8–12 repetitions per set to achieve voluntary fatigue, and do it ≥2 times per week. Exercises for the same muscle group should be separated by at least 1 day (16, 19). A study has shown that, when repeated regularly, short bursts of vigorous intensity PA can result in substantial improvements in cardiorespiratory fitness and other cardiovascular outcomes (31).

### Repetition and substitution

Fourteen studies involve repetition and substitution. To address sedentary behaviors in people at high risk for cardiovascular disease, we can urge the use of beneficial or neutral behaviors to replace undesirable behaviors, such as advocating activity and standing behaviors for a long period of time (18, 20), changing sedentary behaviors to light physical activity or moderate to high physical activity (15, 16, 26), and promoting practice and repetition of alternative behaviors to replace unhealthy behavioral habits. If prolonged healthy behaviors are difficult to adhere to, target behaviors can also be set as small tasks that are simple to difficult and achievable, such as interrupting sedentary activity for 1 h for 8 min of moderate-intensity PA (21), interrupting sedentary activity for 30 min for 2 min of exercise (22), interrupting sedentary activity for 30 min of PA for 1 min of high-intensity PA (26), exercising with a heart rate of 90% of maximum heart rate, and exercising for 45 min after a meal for 20 min of high-intensity PA after a long sedentary period (21). The ultimate goal of the intervention is that health behaviors will eventually become habitual behaviors.

### Regulation

Four studies involve regulation. In order to minimize the development of negative emotions, PA can be integrated into daily life (15). Choosing a single workout may make the whole change boring and make it difficult to stick to. However, the guidelines have given advice on different workouts and there are studies that show that PA is synergistic with each other. For example, running and cycling, running and aerobics, running and racquetball (29).

### Antecedents

Eight studies involve antecedents. Creating and altering the physical environment around a research subject can create barriers to unhealthy behaviors, such as changing the workplace environment (20, 25, 30, 32). Avoiding exposure or contact with social environments that can cause specific unhealthy behaviors and advocating for changes in the environment of the communities in which we live (32).

### Identity

Two studies involve identity. Proposing to the research subjects to be proactive in accepting new perspectives for understanding the target behavior and changing their perceptions about implementing health behaviors makes the research subjects notice the differences between current and past behaviors and perceptions (24, 25), which in turn better facilitates behavioral change.

### Self-belief

One study involves self-belief. The intervention process should build the confidence of the subject, encouraging him to be sure that he can do it and will succeed, thus reinforcing the whole training process (32). Self-confidence is an internal thing, have the intervention subject imagine successful behavior change in the scenario in question, encouraging positive self-talk throughout.

## Discussion

This scoping review aimed to identify and evaluate the behavior change techniques to reduce sedentary behavior and increase physical activity in people at high risk for cardiovascular disease and to improve the effectiveness of interventions, and retrospectively code the BCTs employed in identified interventions using the BCTTv1 framework. A total of 19 studies were identified for this review that employed different behavioral strategies in the included interventions. However, of the 16 groups of BCTTv1 categories, only 11 were identified in 18 studies. This finding suggests that there are other behaviors that are currently unexplored in people at high risk for cardiovascular disease and that these strategies may prove effective in improving SB and PA in this population.

The outcomes of metabolic measures of cardiovascular disease risk extracted from the included studies showed that each type of behavioral intervention could be effective in reducing cardiovascular disease risk in people at high risk (21, 24, 30, 34). However, beyond behavioral techniques that directly intervene in sedentary behavior or physical activity, there are some important considerations worth noting. Direct behavioral interventions have resulted in significant changes in cardiovascular risk factors (23). However, when cultural context is considered, implementing direct behavioral interventions without incorporating cultural factors presents notable challenges (24). This idea is consistent with the Culture-Behavior-Brain loop model (35). The theory states that culture can not only change behavior, but even influence future brain changes through behavior. For instance, there was a study conducted in the UK that utilized text messages related to cultural content to influence the dietary and physical behaviors of postpartum mothers (36). In the future, more culturally-integrated intervention measures should be carried out.

Many guidelines and studies have recommended the intensity of physical activity and the time to break sedentary behavior (16, 17). Some studies have even proposed moderate-to-vigorous physical activity during interruptions in sedentary behavior (22, 26). However, the feasibility of implementation of this series of interventions warrants consideration. For instance, interventions targeting the workforce population. Among the studies included, one was an intervention to reduce sedentary behavior in the workplace (30), and the other was to increase physical activity among female migrant workers in the workplace (34). Although the cardiac risk indicators were reduced after the intervention, the future persistence and compliance of the intervention subjects were not involved. Because frequent breaks from prolonged sitting can reduce work efficiency, and whether the body can withstand the moderate-intensity physical activities during the interruption period is also worth exploring. For the working population, we advocate adopting a flexible approach and integrating PA into daily life (15). Sporadic brief bursts of vigorous physical activity in daily life have been shown to benefit cardiovascular health (31).

Numerous guidelines and studies have provided recommendations for the type and frequency of PA (1, 16, 29). Some studies have even shown that different types of PA have different effects on cardiovascular risk (29). For example, American football is the most beneficial sport, while swimming and racquet sports have no effect on cardiovascular risk (29). However, this study was a cross-sectional survey of the population, and the duration and intensity of exercise were not clear. Future studies should control more interference factors to compare PA types. Resistance exercise has been recommended by more and more guidelines in recent years (19). There are studies showing that resistance exercise can increase muscle mass, strength, and physical function (37). In the future, more in-depth research can be conducted on resistance exercises.

While the rationale behind the inclusion of specific BCTs within studies cannot be determined through retrospective coding, the justification for intervention design lacks clarity, and in certain instances, empirical substantiation. However, the combination of behavior change theory and intervention can significantly improve the intervention effect (32). Various behavioral theories can be applied to the behavior change of the high-risk population of cardiovascular disease, such as the Information-Motivation-Behavioral model (38). Information-Motivation-Behavioral model can be used to identify which of the core components of information, motivation, and behavioral are underpinning the undesired behavior, and can then be mapped onto the BCTTv1 such that only relevant BCTs are incorporated within a precisely targeted intervention (12).

The quality appraisal conducted on the included studies showed that study quality was generally satisfactory, but highlighted some limitations within the current literature. The limitations of randomized controlled trials are that participants were not blinded (20), and outcomes for those who were lost to follow-up were not described and included in the analysis (21). The design and analysis of randomized controlled trials should be more comprehensive to ensure the reliability of the trial. Although there was systematic review in the included literature (30), it only summarized the interventions for the workplace population, and ignored the family, community and other places. And this scoping review included a wider range of factors influencing behavior for discussion. In the future, the design of behavioral research should be more comprehensive and rigorous, and the intervention research should control the implementation conditions as much as possible to ensure the reliability of the research.

### Limitations

Some limitations emerge from this review. First, this review protocol was not registered previously. Second, studies published in English and Chinese were only included in specific databases, thus some relevant studies published in other languages might have been omitted. Lastly, the suboptimal quality of some RCTs highlighted a need for better design and/or reporting of RCTs for interventions aimed at improving adherence. This highlights the necessity for better design and/or reporting of intervention measures aimed at improving compliance. It is essential to provide a transparent and comprehensive description of BCTs and its intervention characteristics to offer clarity for future research. It is recommended that interventionists explicitly indicate BCTs when formulating intervention measures to enhance the accuracy of reporting, understanding, evaluation, and the transmission of effective components. We hope that this review on specific BCTs and the characteristics of successful static behavior and physical activity interventions will be helpful for the development of future intervention measures.

## Conclusion

To summarize, this scoping review aimed to identify and evaluate the behavior change techniques to reduce sedentary behavior and increase physical activity in people at high risk for cardiovascular disease. Although all aspects of the intervention have shown beneficial changes, the durability of the changes is worth exploring. Although moderate-to-vigorous intensity PA has been shown to have cardiovascular benefits, the choice of PA type is still controversial. Furthermore, the limited range of BCTs used would indicate that there are alternative behavioral components that have yet to be examined in people at high risk for cardiovascular disease. Moving forward, future studies should carry out more comprehensive interventions on behavior, such as PA types, cultural backgrounds,which can make the intervention more reliable. Furthermore, any behavioral components incorporated into interventions should be specified using standardized terminology, such as those outlined in the BCTTv1 to implement targeted interventions for behaviors.

## Data Availability

The original contributions presented in the study are included in the article/Supplementary Material, further inquiries can be directed to the corresponding authors.
